# Commissioning kilovoltage cone‐beam CT beams in a radiation therapy treatment planning system

**DOI:** 10.1120/jacmp.v13i6.3971

**Published:** 2012-11-08

**Authors:** Parham Alaei, Emiliano Spezi

**Affiliations:** ^1^ Department of Radiation Oncology University of Minnesota Minneapolis MN 55455 USA; ^2^ Department of Medical Physics Velindre Cancer Centre Cardiff UK

**Keywords:** cone‐beam CT, kilovoltage, treatment planning

## Abstract

The feasibility of accounting of the dose from kilovoltage cone‐beam CT in treatment planning has been discussed previously for a single cone‐beam CT (CBCT) beam from one manufacturer. Modeling the beams and computing the dose from the full set of beams produced by a kilovoltage cone‐beam CT system requires extensive beam data collection and verification, and is the purpose of this work. The beams generated by Elekta X‐ray volume imaging (XVI) kilovoltage CBCT (kV CBCT) system for various cassettes and filters have been modeled in the Philips Pinnacle treatment planning system (TPS) and used to compute dose to stack and anthropomorphic phantoms. The results were then compared to measurements made using thermoluminescent dosimeters (TLDs) and Monte Carlo (MC) simulations. The agreement between modeled and measured depth‐dose and cross profiles is within 2% at depths beyond 1 cm for depth‐dose curves, and for regions within the beam (excluding penumbra) for cross profiles. The agreements between TPS‐calculated doses, TLD measurements, and Monte Carlo simulations are generally within 5% in the stack phantom and 10% in the anthropomorphic phantom, with larger variations observed for some of the measurement/calculation points. Dose computation using modeled beams is reasonably accurate, except for regions that include bony anatomy. Inclusion of this dose in treatment plans can lead to more accurate dose prediction, especially when the doses to organs at risk are of importance.

PACS numbers: 87.55.D, 87.55.K, 87.56.bd

## I. INTRODUCTION

Cone‐beam CT (CBCT) is rapidly becoming the standard equipment in radiation therapy departments employing image‐guided radiation therapy (IGRT) for patient position verification. Cone‐beam CT acquisition is performed either using the linear accelerator's megavoltage beam (MV CBCT) or an additional kilovoltage beam (kV CBCT).

Considering the frequency of these imaging procedures and the magnitude of the dose, calculation and accounting of the dose from CBCT and other imaging procedures is of interest.^1^, ^8^

There have been many studies on the dose delivered to patients from CBCT procedures. Among the studies that have focused on kilovoltage CBCT dose, Islam et al.^(2)^ measured doses in phantom on an Elekta X‐ray volume imaging (XVI) unit and reported doses of up to 3.5 cGy per procedure. Kan et al.^(5)^ made similar measurements on a Varian on‐board imager (OBI) unit and reported doses ranging from 4–6 cGy to the involved organs. Song et al.^(6)^ measured doses for both Elekta and Varian units, and reported doses of up to 3.5 cGy for the Elekta and 8.3 cGy for the Varian unit. These investigations focused on the systems available at the time, and current systems may result in less dose than stated.

There have also been extensive studies done on calculating the CBCT dose using Monte Carlo (MC). Among these, Ding et al.^(9)^ simulated dose distribution from Varian OBI unit and calculated the dose to several organs as a result of imaging procedures. Spezi et al.^10^, ^11^ and Downes et al.^(12)^ carried out a full dosimetric characterization of the Elekta XVI unit and simulated three‐dimensional concomitant dose distributions in various anatomical regions.

Due to the limitations of the treatment planning systems and their algorithms (which are primarily designed for megavoltage beams), including the dose in the radiation therapy treatment plans is not easily achievable. In previous work, the Varian half‐bowtie half‐fan CBCT beam was modeled in a commercial treatment planning system.^(13)^ That work was limited to only one beam quality and one acquisition protocol. The present work focuses on full commissioning of the CBCT beams from the Elekta XVI system in a commercial radiotherapy treatment planning system (TPS). The commissioning includes multiple beams with various technique, field sizes (cassettes), and filter settings. The modeling of the depth‐dose and cross profiles, as well as measurement of output factors for various beams, is presented here. This work also includes dose calculations in a simple stack phantom, as well as in three regions of an anthropomorphic phantom representing anatomic locations commonly encountered in radiation therapy. Extensive measurements and Monte Carlo simulations have also been performed to evaluate degree of accuracy of the method.

## II. MATERIALS AND METHODS

In this work, we have commissioned the kV CBCT beams from the Elekta XVI system (Elekta, Atlanta, GA) in a commercial treatment planning system (Pinnacle, Philips Medical Systems, Milpitas, CA), computed the dose to phantom using this system, and compared the results to TLD measurements and Monte Carlo simulations. The Elekta XVI system has been previously described.^(10)^ In summary, it employs, in its default clinical presets, 100 and 120 kVp beams and a range of cassettes which determine the imaging field size. The cassettes are named based on the imaging field of view (small (S), medium (M), and large (L)) and the length of the imaging field (2, 10 or 20 cm). The imaging can be done without any additional filtration (denoted as F0), or with the addition of a bowtie filter (denoted as F1). Imaging techniques (kVp, mA, ms, and gantry start/stop angles) are preset, depending on the anatomical site being imaged and the presence of bowtie filter. These clinical presets can be modified by the user.

### A. Beam modeling

The treatment planning system (TPS) used in this study is a commercial product (Pinnacle v8.0m), but with the addition of low‐energy deposition kernels (20–110 keV) to extend its computational capabilities to kilovoltage range. Although the energy range of the kernels does not cover the full spectrum of energies possible in a 100 or 120 kVp beam, modeling of a beam with beam qualities desired here is easily achievable with the available kernels. The generation, validation, and use of these low‐energy deposition kernels has been described previously.^14^, ^16^

The beam data required for modeling have been measured and verified using Monte Carlo by Spezi et al.^(10)^

The data used in the TPS commissioning process is listed in Table 1. These data, acquired for a combination of cassettes/filters and beam qualities, include depth‐dose curves and cross profiles at 1, 5, and 10 cm depths for each beam. Measured CBCT profiles were integrated with MC‐generated profiles when needed.

**Table 1:** Elekta XVI CBCT beams/techniques for which the beam data were collected and modeling performed, and the results of output factor measurements (in phantom at 1 cm depth with 100 cm SSD) for these beams.

*Cassette*	*Filter*	*kVp*	*mA*	*ms*	*Output (cGy/min.)*
S10	F0	100	10	10	0.20
S20	F0		10	10	0.20
S20	F0	120	40	40	6.00
M20	F0		25	40	3.55
	F1		40	40	4.40
M10	F0		40	40	5.60
L20	F0		10	25	0.80
	F1		64	40	6.40
L10	F0		10	25	0.80

Beam output factors were also measured for these beams. Output measurements were performed using an Exradin A12 ionization chamber (Standard Imaging, Madison, WI) in a Certified Therapy Grade (CTG) solid water phantom (Gammex RMI, Middleton, WI). This solid water material is composed of hydrogen, carbon, nitrogen, oxygen, chlorine, and calcium with a physical density of 1.043 g/cc. The ionization chamber was calibrated using a similar beam quality at an accredited calibration laboratory. The measurements were performed at 1 cm depth in phantom, with a source‐to‐surface distance (SSD) of 100 cm and 7 cm backscatter using a static beam delivering 200 image frames. The use of solid water in kilovoltage X‐rays has been examined by Hill et al.^(17)^ and found to be accurate, compared to water, to within 1%.

The AAPM Task Group 61 protocol^(18)^ was used to compute the absorbed dose from ionization. The output measurements were repeated several times on the same unit and found to be stable to within 1% in a span of a few weeks. There is slightly larger variation in output over a span of months. The results of output factor measurements and the techniques used are also listed in Table 1. These output factors were entered in the system as cGy/MU, with 1 MU being effectively equivalent to 1 minute. The choice of time would make it possible to enter acquisition time for a particular imaging study in lieu of monitor units in the prescription.

Upon import of the data into the TPS, both auto and manual modeling were employed to obtain the best fit to the measured data. In the case of the cassettes with an offset (M and L) and/or those with bowtie filter, a beam modifying device, defined as a wedge in Pinnacle, was inserted in each beam to alter the modeled beam profiles to match the measured ones. These beam modifiers are part of the beam model and must be included in the respective beams for all dose calculations.

### B. Validation of modeling

**Figure 1:**
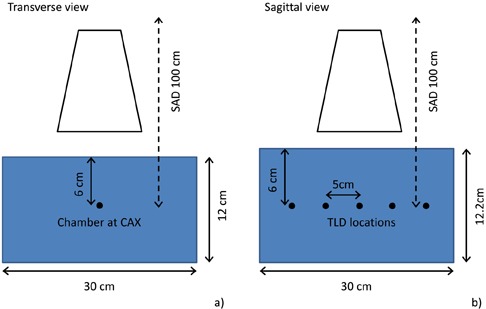
The diagram of the solid water stack phantom including the locations of the ion chamber (a) and TLD chips (b).

In order to validate the beam modeling and dose computation by Pinnacle, two phantoms were employed. The first phantom consisted of a 12 cm thick stack of 30 by $30\;{\text{cm}}^{2}$ CTG solid water with the same Exradin A12 ionization chamber in the center ((Fig. 1a).

The second phantom consisted of a 12.2 cm stack of 30 by $30\;{\text{cm}}^{2}$ solid water. A 2 mm slab of solid water with five milled slots, with 5 cm spacing for placement of TLDs, was used in the center of phantom in the superior–inferior direction ((Fig. 1b). The TLD chips were placed in the five milled slots. The two phantoms were scanned using a Philips Brilliance CT scanner (Philips Healthcare, Andover, MA) and imported into the TPS. Six arc beams of 360° each, representing the M20 (F0 and F1), S20 (100 kVp), L20 (F0 and F1), and M10 (F0) cassettes, were placed on both phantoms and the dose from each beam was computed. The grid resolution used for dose computation was 0.3 by 0.3 by $0.3\;{\text{cm}}^{3}$. The treatment couch was simulated in the TPS by inserting a slab under the phantoms and assigning appropriate density to it to produce the level of attenuation of the beam by the couch, based on the couch attenuation measurements previously done with the same quality beam. The locations of ion chamber and TLD chips were contoured on the phantom images to obtain computed doses.

The same six arc beams were delivered to the phantoms and the delivered dose was measured using the A12 ionization chamber in the first phantom configuration ((Fig. 1a) and TLD‐100 chips in the second ((Fig. 1b). TLD irradiation, reading, and annealing were performed following the same protocol used for their calibration, as described below in Materials Section D.1. The actual number of frames acquired for each delivery was recorded in each case. These measurements were performed four times each. For the low‐dose beams (S10, F0, 100 kVp, and L20, F0, 120 kVp) each measurement constituted multiple deliveries, 10 for the S10 and 3 for the L20, in order to deliver high enough doses to get reliable readings. The data from each acquisition were used to calibrate the MC simulation, as described below in Materials Section E.

### C. Dose computation

Three of the validated kV CBCT beams were used to compute the dose to an anthropomorphic phantom. The RANDO phantom (The Phantom Laboratory, Salem, NY, USA) was scanned using a Philips Brilliance CT scanner and the images were imported into Pinnacle treatment planning system. CBCT acquisitions were simulated by placing arc beams on three locations: head and neck, chest, and pelvis. Approximating a CBCT delivery, with its many frames, in a planning system using arcs has been previously discussed.^(13)^ The treatment couch was inserted under the phantom, as described above. Beam arrangements are listed in Table 2. The locations of TLD chips were contoured on the phantom image (similar to above) to obtain computed doses. The grid resolution used for dose computation was 0.3 by 0.3 by $0.3\;{\text{cm}}^{3}$ for this phantom, as well.

**Table 2:** The beam travel range and cassette/filter/energy used in CBCT acquisition and the beam arrangements used for dose calculation in the RANDO phantom. The time is the actual acquisition time as measured on the unit.

*CBCT Acquisition on the XVI Unit*						*CBCT Reproduction in Planning System*		
*Site*	*Cassette*	*Filter*	*Energy*	*kV tube start/stop angle*	*Time (min.)*	*Arc*	*Start angle*	*Stop angle*
Head and Neck	S20	F0	100 kVp	345/190	1	HN Arc	345	190
Chest	M10	F0	120 kVp	270/270	2	Chest Arc	271	270
Pelvis	M20	F1	120 kVp	270/270	2	Pelvis Arc	271	270

### D. Dose measurements

The CBCT beams listed in Table 2 were delivered to the RANDO phantom and dose was measured at various points using LiF thermoluminescent dosimeters (TLD‐100) chips (Thermo Fisher Scientific, Inc. Waltham, MA). The TLD chips were placed inside the phantom at predrilled holes and secured in place by Mix D plugs supplied with the phantom. TLD irradiation, reading, and annealing were performed following the same protocol used for their calibration, as described in Materials Section D.1. These measurements were performed three times. In the case of head‐and‐neck acquisition, each measurement included 15 consecutive acquisitions of the CBCT, in order to deliver a measurable dose.

#### D.1 TLD measurements

Due to the energy dependence of TLD‐100, it was deemed necessary to calibrate the TLD chips using the same beam quality as that used for measurements. Hence, 30 TLD‐100 chips were calibrated according to the following procedure. The TLD chips were first annealed at 400°C for one hour and were left to cool down. They were then transferred to a polyethylene holder with inserts large enough to house TLD chips in place with minimum air gap. The XVI unit's output was measured at 1 cm depth in solid water using an ionization chamber, and the TLD chips were subsequently placed at 1 cm depth and irradiated with a known dose (based on ionization chamber measurements). The TLDs were read after 40 hours using a Harshaw model 3500 TLD reader (Thermo Fisher Scientific, Inc.). This was performed three times. The constancy of response of TLDs was evaluated and chips with a response variation of greater than 3% were not used. A calibration factor (cGy/nC) was thus obtained for each chip and used to convert TLD signal to dose for phantom measurements.

### E. Monte Carlo simulations

The CT scan data of both stack and anthropomorphic phantoms and all plan information for the cases are discussed in Sections A & B above and in Table 2, and were prepared in Pinnacle, exported as RTOG files, and processed for Monte Carlo (MC) simulation. The calculation engine used in this work was DOSXYZNRC,^(19)^ which is part of the EGSNRC MC Code system^(20)^ version V4‐r2‐2‐5. A library of prebuilt and validated phase space (PHSP) files was used to simulate the RANDO phantom irradiation using the XVI unit. The MC transport geometry was built from the phantom CT scan using a CT‐to‐Hounsfield Unit conversion function including air, lung, tissue, and bone. Foam and carbon materials were also used to simulate the carbon fiber couch top. The MC dose calculation grid was 0.2 by 0.2 by $0.3\;{\text{cm}}^{3}$. For the energies considered in this investigation, electrons produced in the body were absorbed locally in the voxel in which they were generated. Hence, the energy thresholds for secondary particle transport were set to the EGSnrc default of ECUT=0.700 MeV for electrons and PCUT=0.01 MeV for photons. To increase the efficiency of dose calculation, a photon‐splitting number n_split=100 was used. The statistical uncertainty associated with the calculations varied between ±2% for chest and pelvis and ±3% for head and neck (1 standard deviation).

Absolute dose calculation was carried out by calibrating the MC output in terms of Gy/incident particle/frame with an approach similar to the one described by Downes et al.^(12)^ In this case, the dose delivered at the center of a 12 cm thick solid water phantom was measured with an Exradin A12 ionization chamber for the CBCT presets used with the RANDO phantom. Both TPS and MC dose matrices, segmented volumes of interest (VOIs), and plan information were loaded into and independently analyzed with the Computational Environment for Radiotherapy Research (CERR) and in‐house software^21^, ^22^ developed in the MATLAB environment (The MathWorks, Natick, MA, USA).

## III. RESULTS & DISCUSSION

### A. Beam modeling and validation

**Figure 2:**
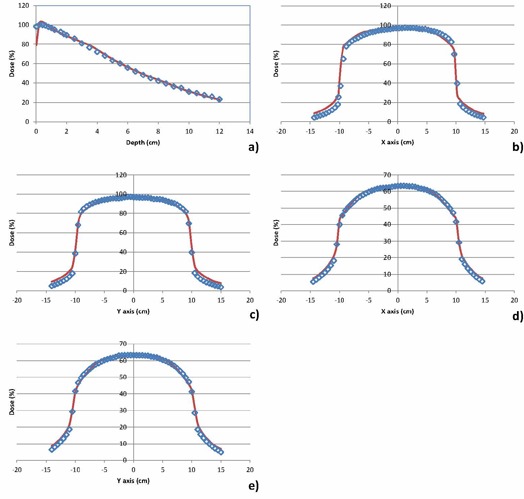
Comparison of measured and modeled depth‐dose and cross profiles for 100 kVp beam with S20 cassette: a) depth dose; b) X profile at 1 cm depth; c) Y profile at 1 cm depth; d) X profile at 5 cm depth; e) Y profile at 5 cm depth. The points represent measured values and the solid lines modeled profiles. The percentage difference between measured and computed doses is 1.7% for the depth dose and between 1.6 and 3.8% for the cross profiles.

**Figure 3:**
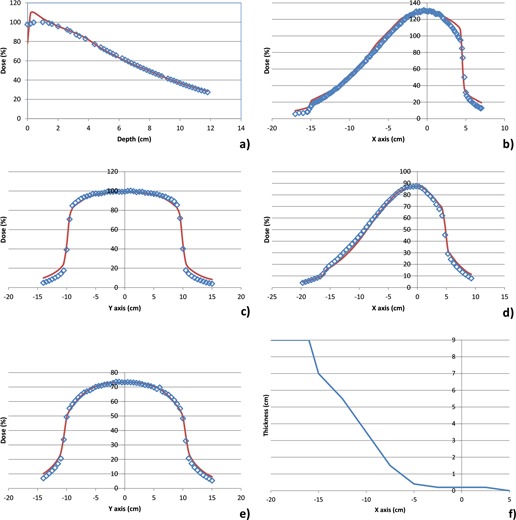
Comparison of measured and modeled depth‐dose and cross profiles for 120 kVp beam, M20 cassette, with bowtie filter: a) depth dose; b) X profile at 1 cm depth; c) Y profile at 1 cm depth; d) X profile at 5 cm depth; e) Y profile at 5 cm depth; f) beam modifier (wedge) profile used to shape modeled beam to fit measured data. The points represent measured values and the solid lines modeled profiles. The percentage difference between measured and computed doses is 1.4% for the depth dose and between 1.9 and 3.8% for the cross profiles.

**Figure 4:**
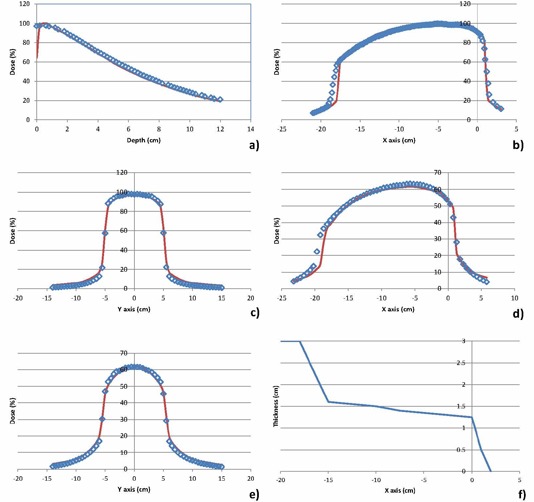
Comparison of measured and modeled depth dose and cross profiles for 120 kVp beam, L10 cassette, without bowtie filter: a) depth dose; b) X profile at 1 cm depth; c) Y profile at 1 cm depth; d) X profile at 5 cm depth; e) Y profile at 5 cm depth; f) beam modifier (wedge) profile used to shape modeled beam to fit measured data. The points represent measured values and the solid lines modeled profiles. The percentage difference between measured and computed doses is 2.6% for the depth dose and between 2.0 and 3.1% for the cross profiles.

The modeled depth‐dose and cross profiles for three of the beams are shown in Figs. 2–4. Figure 2 shows the modeled beam for the 100 kVp beam, S20 cassette without a filter, Fig. 3 shows the modeled beam for the 120 kVp beam, M20 cassette with the bowtie filter, and Fig. 4 shows the modeled beam for the 120 kVp beam, L10 cassette without a bowtie filter. The beam modifiers used to account for beam asymmetry and/or bowtie are also shown in Figs. 3 and 4. The depth dose values generally agree to within 2% at depths beyond 1 cm, with the average absolute percentage difference between measured and computed values ranging from 1.4% to 2.6%. Percentage difference is calculated as: $((\text{computed}‐\text{measured})/D_{\text{max}})*100$.

The cross profiles at three depths (1, 5, and 10 cm) and in two directions (X, Y) generally agree to within 2% in regions within the field, with larger discrepancies observed in the beam penumbra. The average absolute percentage difference between measured and computed values in all regions of the beams range from 1.6% to 3.8%. Percentage difference is calculated as: ((computed‐measured)/central axis dose)*100.

**Figure 5:**
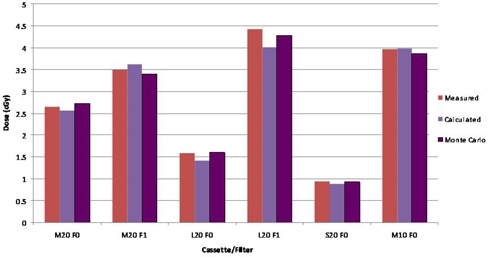
Results of comparison of TPS‐computed doses, ion chamber measurements, and Monte Carlo simulations made in phantom depicted in (Fig. 1a).

**Figure 6:**
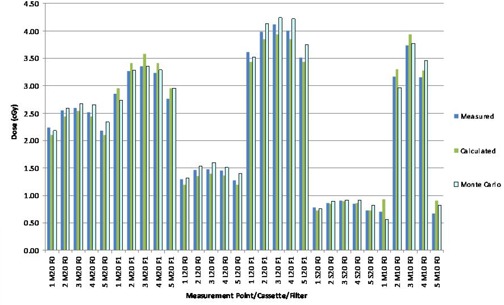
Results of comparison of TPS‐computed doses, TLD measurements, and Monte Carlo simulations made in phantom depicted in (Fig. 1b).

The results of computing dose to the stack phantoms and comparison with measured and Monte Carlo‐calculated doses are shown in Figs. 5 and 6. The TPS‐calculated values are the mean dose to each contoured TLD or ionization chamber location. An analysis of data in Fig. 5 indicates an average percentage difference of 5.16% between measured and TPS‐computed values and an average 3.55% difference between Monte Carlo and TPS‐computed values for the ion chamber measurements. Similar analysis for TLD measurements (Fig. 6) indicates an average percentage difference of ‐0.2% between measured and TPS‐computed values and 2.85% between Monte Carlo‐ and TPS‐computed values. Larger variances are observed for the ion chamber measurements for the two L cassettes. The increased discrepancy for the L cassette is due to the off‐axis aperture of the cassette, which places the point of dose measurement with ion chamber in regions near or within the beam penumbra where the agreement between measured and modeled profiles is the least. Also, the two TLD measurements at low dose points show large differences from calculated values.

### B. Dose computation and verification

**Figure 7:**
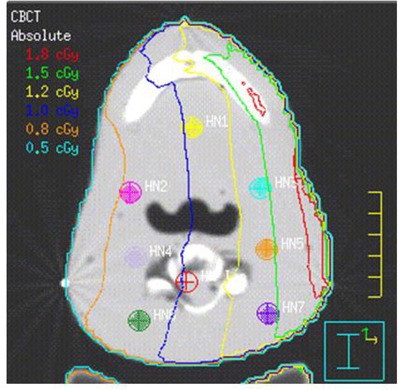
Axial CT slice of the RANDO phantom at the location of isocenter and TLD placement for the head‐and‐neck CBCT acquisition listed in Table 2. TLD positions refer to those listed in Table 3. Isodose lines listed are for 15 acquisitions.

**Figure 8:**
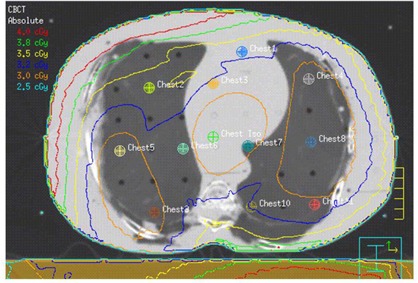
Axial CT slice of the RANDO phantom at the location of isocenter and TLD placement for the chest CBCT acquisition listed in Table 2. TLD positions refer to those listed in Table 3. Isodose lines listed are for 1 acquisition.

**Figure 9:**
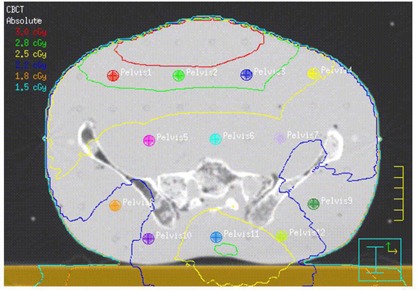
Axial CT slice of the RANDO phantom at the location of isocenter and TLD placement for the pelvis CBCT acquisition listed in Table 2. TLD positions refer to those listed in Table 3. Isodose lines listed are for 1 acquisition.

**Figure 10:**
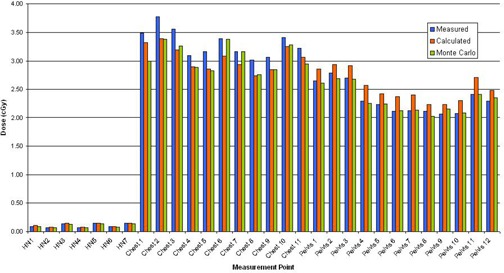
Results of comparison of TPS‐computed values, TLD measurements, and Monte Carlo simulations made in RANDO phantom. Listed locations refer to data in Table 3 and depicted in Figs. 7–9.

The locations of TLDs on the CT images of the RANDO phantom for all three CBCT acquisitions are shown in Figs. 7–9. In each case, the mean dose to each contoured TLD location was obtained from the planning system. The results of comparison of measured and MC‐computed values to the TPS‐computed ones for each TLD are tabulated in Table 3 and shown in Fig. 10. As noted in the Materials Section D above, the head‐and‐neck dose measurements and computations were performed for 15 acquisitions and the dose for a single acquisition was extracted from this. An analysis of Table 3 results indicates an average percentage difference of ‐2.15% between measured and computed values, with a standard deviation of 8.97 and average percentage difference of ‐8.75% between Monte Carlo and computed values, with a standard deviation of 6.03, indicating an over‐estimation of the dose by the planning system. Analyzing the data individually per beam/site, the percentage differences in the head‐and‐neck and pelvic regions follow the same trend, whereas those of the chest show an opposite trend (i.e., planning system underestimating the dose). There are individual points exhibiting large percentage differences. These are partially due to the fact that small variations in low doses translate into large local percentage differences, and that some of these are located in and near bony heterogeneities, which add to the uncertainty of dose calculations both due to the limitations of dose calculation algorithm and the potential lack of electronic equilibrium. But overall, it can be concluded with certainty that the planning system calculates the dose to within 10% of actual dose, except in bone.

**Table 3:** Comparison of measured, MC‐computed and TPS‐computed doses in the three CBCT acquisitions in RANDO phantom. All doses listed are for one acquisition and are in cGy. Percent differences are computed as: ((Measured‐Computed)/Computed))^*^100 and ((Monte Carlo‐Computed)/Computed))^*^100.

*Measurement Location*	*TPS‐Computed Dose*	*Measured Dose*	*MC‐Computed Dose*	*Meas. vs. TPS % Difference*	*MC vs. TPS % Difference*
Head and Neck					
HN 1	0.105	0.087	0.087	‐16.84	‐16.84
HN 2	0.078	0.065	0.069	‐16.28	‐11.12
HN 3	0.144	0.137	0.132	‐4.52	‐8.33
HN 4	0.082	0.071	0.073	‐13.09	‐10.58
HN 5	0.143	0.143	0.135	0.21	‐5.81
HN 6	0.089	0.087	0.083	‐2.27	‐6.78
HN 7	0.145	0.144	0.138	‐0.73	‐4.72
			Average:	‐7.65	‐9.17
Chest					
Chest 1	3.32	3.49	2.99	5.10	‐9.86
Chest 2	3.39	3.78	3.38	11.44	‐0.31
Chest 3	3.19	3.56	3.27	11.50	2.35
Chest 4	2.90	3.09	2.89	6.56	‐0.38
Chest 5	2.86	3.16	2.82	10.54	‐1.27
Chest 6	3.08	3.39	3.38	10.20	9.70
Chest 7	2.94	3.16	3.16	7.58	7.63
Chest 8	2.74	3.02	2.76	10.19	0.62
Chest 9	2.85	3.06	2.85	7.43	‐0.17
Chest 10	3.25	3.41	3.28	4.89	0.90
Chest 11	3.06	3.22	2.95	5.18	‐3.76
			Average:	8.24	0.49
Pelvis					
Pelvis 1	2.86	2.65	2.61	‐7.47	‐8.69
Pelvis 2	2.94	2.79	2.69	‐5.03	‐8.38
Pelvis 3	2.92	2.70	2.69	‐7.63	‐8.06
Pelvis 4	2.57	2.30	2.26	‐10.60	‐12.11
Pelvis 5	2.42	2.24	2.25	‐7.58	‐7.04
Pelvis 6	2.37	2.12	2.13	‐10.51	‐10.32
Pelvis 7	2.40	2.13	2.14	‐11.33	‐11.05
Pelvis 8	2.24	2.12	2.02	‐5.43	‐10.05
Pelvis 9	2.24	2.06	2.16	‐7.85	‐3.73
Pelvis 10	2.31	2.08	2.09	‐9.93	‐9.52
Pelvis 11	2.71	2.42	2.42	‐10.77	‐10.88
Pelvis 12	2.48	2.30	2.35	‐7.45	‐5.19
			Average:	‐8.46	‐8.75

## IV. CONCLUSIONS

Computing and adding CBCT dose to patient treatment plans is the subject of discussion in the radiation therapy community. Addition of the dose from kilovoltage CBCT is currently not possible in clinical settings due to the inability of treatment planning systems to accurately model and compute this dose. This investigation examined the possibility of modeling and commissioning the beams produced by a kV CBCT system using a commercial treatment planning system and the degree of accuracy of such a dose calculation. Even though the calculation accuracy of currently available TPS algorithms developed for MV dose calculations is still limited in regions with a density significantly different than that of soft tissue, the 3D calculation of concomitant dose could still provide clinically adequate information, especially for estimating critical additional dose to the organs at risk. Accounting for the imaging dose would help the clinicians to make better decisions during treatment planning process, and would be a useful step towards a fuller implementation of adaptive radiotherapy for the increased benefit of the patient.

In this study, we have commissioned a commercial TPS, with extended capabilities to compute the dose in the kV range, for CBCT dose calculations. This system can now be used to compute the dose from kV CBCT and to add it to the dose received from radiation therapy. Adding the CBCT beam is as simple as adding an arc to the treatment plan. The time to compute the dose from this arc depends on the technical specifications of the workstation and on the resolution of the calculation grid. A full three‐dimensional dose calculation takes up to 30 minutes on an 810x workstation (Oracle, Redwood City, CA) with a 2.8 GHz Intel Xeon quad‐core CPU and 16 GB of RAM. However, this calculation can be done in parallel with other planning activities and could also be automated using a script. Hence, the additional planning time could be efficiently minimized and its implementation in routine clinical practice would be feasible.

As described in this investigation, the implementation of CBCT dose calculation in a commercial TPS requires measurements and beam modeling routines specific for the kV beams. Currently, the acquisition of such additional data is not part of default TPS commissioning protocols. Moreover, standard beam modeling tools, as implemented in commercial systems, are not designed to process kV beam data. Therefore, more work by the manufacturers is needed to provide end users with the capability of including kV CBCT dose into the treatment planning process.

During this work, we had the opportunity to compare the beam data from three Elekta XVI systems, two in the United States, and one in the United Kingdom, and found a match at the 2%/2 mm level for both depth doses and profiles. Although these data are preliminary, they led us to believe that beam models developed in one institution could be used as template in multiple clinics. This would facilitate the commissioning process and reduce the amount of data required for the CBCT commissioning to a small set of verification measurements.

As for the accuracy of TPS algorithms in the kV range, current and future work shall provide more accurate algorithms to compute the dose to bone in this energy range. For example, Monte Carlo‐based commercial treatment planning algorithms could provide accurate dose computation from kV sources as the CBCT source models are developed.^23^, ^24^ There has also been some work on development of algorithms to compute dose accurately in the kV range, an example of which is the work done by Ding et al.^(25)^

Future work in this area includes a clinical study investigating the feasibility of adding CBCT dose to patient treatment plans on a routine basis.
